# A novel 5-stage visual rating scale for global arterial spin labeling perfusion assessment in the brain: Simplifying evaluation for clinical implementation

**DOI:** 10.1016/j.cccb.2024.100200

**Published:** 2024-01-02

**Authors:** Emilian Kalchev, Radoslav Georgiev, Darina Ivanova

**Affiliations:** a Department of Diagnostic Imaging, St Marina University Hospital, Varna, Bulgaria; b Department of Diagnostic Imaging, Interventional Radiology and Radiotherapy, Medical University of Varna, Bulgaria

**Keywords:** Magnetic resonance imaging, Arterial spin labeling (ASL), Cerebrovascular circulation, Cerebral small vessel disease

## Abstract

**Objectives:**

The aim of this study was to develop and validate a visual rating scale for evaluating global arterial spin labeling (ASL) perfusion changes in the brain, with potential applications in a variety of conditions that impact general brain blood supply and perfusion.

**Methods:**

We employed a five-stage scale (0 being normal and 4 indicating the most severe perfusion decline) to assess 156 patients using a 3D pulsed ASL technique. Three radiologists independently reviewed the images, and inter-rater reliability of the visual rating scale was evaluated.

**Results:**

The ASL stages showed a consistent distribution among the patients. The inter-rater reliability among the three radiologists, as measured by the Intraclass Correlation Coefficient (ICC), was 0.982.

**Conclusion:**

Our findings suggest that this visual rating scale can be effectively implemented in everyday practice to evaluate global perfusion changes in the context of cardiovascular diseases, cerebrovascular diseases, cerebral small vessel disease, and other conditions that alter brain vascularization and perfusion. Further research is needed to explore the full range of clinical applications and to refine the scale for optimal utility.

## Highlights

• Utilized arterial spin labeling (ASL) for a non-invasive brain perfusion assessment.
• Developed a novel 5-stage visual rating scale for ASL changes.
• Demonstrated a high inter-rater reliability of the ASL scale.
• Identified a progressive pattern of perfusion decline across stages.
• The scale is applicable to diverse neurological conditions, including vascular dementia and neurodegenerative disorders.

## 1 Introduction

Arterial spin labeling (ASL) is a non-invasive magnetic resonance imaging (MRI) technique that allows for the assessment of brain perfusion without the need for exogenous contrast agents. Understanding global perfusion changes is crucial, as they can offer insights into cerebral circulation dynamics, which are closely tied to cognitive and behavioral functions. Various factors, ranging from physiological conditions such as age and cardiac output [1], lifestyle choices, mental states [2], to neurological disorders like dementia with Lewy bodies [3], can influence these perfusion patterns. Such diverse sources of variation underscore the importance of a practical method to evaluate global changes in cerebral blood supply and perfusion. ASL has been widely used in the evaluation of these conditions, such as cerebrovascular diseases, neurodegenerative disorders, and more [4,5].

Despite its clinical utility, there is currently no standardized visual rating scale for assessing global perfusion changes in the brain using ASL. This void often leads to inconsistencies in interpretation, especially across varied clinical settings and among radiologists with different expertise levels. Such inconsistencies can potentially affect patient management decisions and the overall understanding of cerebral perfusion dynamics. Visual rating scales can facilitate the interpretation of ASL data by providing a systematic and reproducible method for the assessment of brain perfusion. Moreover, a standardized rating scale could be beneficial for radiologists with varying experience in ASL interpretation and improve the overall consistency of ASL data interpretation across different clinical settings.

In this study, we aimed to develop a novel 5-stage visual rating scale for the assessment of global ASL perfusion changes in the brain. This scale was designed for easy implementation in everyday clinical practice, making it accessible to radiologists with different levels of experience in ASL interpretation.

## 2 Methods

### 2.1 Participants

We analyzed a cohort of 156 patients (ages 45–84) who underwent ASL MRI examinations at our institution. This study was a retrospective analysis of anonymised patient data. In accordance with institutional guidelines, ethics committee approval was not required for this type of study.

Patients were selected from our institution's database, adhering strictly to the defined criteria, as detailed below to ensure that only relevant and quality data was included for analysis. In our selection process, we specifically included participants aged 45 and over, encompassing the categories of middle adulthood (45–59 years) and late adulthood (60 years and above) as defined by the World Health Organization. The choice of this age range was intended to capture a diverse set of perfusion patterns, given the expected varied rates of perfusion decline in these age categories.

Exclusion criteria were defined for:

Conditions known to considerably alter perfusion patterns:
Significant stroke lesions, which can modify regional perfusion.
Brain tumors of substantial size potentially affecting surrounding blood flow.
Extensive cystic changes post-brain surgery, capable of interfering with normal perfusion patterns.
Age below 45 years.
Low-quality images that hampered clear interpretation.

Out of the 156 patients, comprising 101 females and 55 males, 50 had no discernible MRI pathologies. The remaining 106 patients had certain pathological findings. Notably, many exhibited signs of cerebral small vessel disease (CSVD), evidenced by features such as white matter hyperintensities, lacunas, microhemorrhages, or an increased number of dilated perivascular spaces in the basal ganglia. While most of these were of low-grade, some patients had high-grade CSVD changes.

### 2.2 ASL imaging protocol

All MRI examinations were performed on a 3 Tesla MRI scanner (Magnetom Verio, Siemens Healthcare) using a 3D pulsed ASL technique. This technique allowed for only qualitative evaluation and employed a fixed inversion time, which simplified the interpretation and facilitated the implementation in everyday clinical practice [2]. The imaging parameters were as follows: repetition time (TR) = 8000 ms, echo time (TE) = 16.6 ms, inversion time (TI) = 1990 ms, and a voxel size of 3 × 3 × 3 mm^3^.

The MRI protocol also included T1-weighted imaging (T1WI), T2-weighted imaging (T2WI), diffusion-weighted imaging (DWI), fluid-attenuated inversion recovery (FLAIR), and susceptibility-weighted imaging (SWI) sequences.

### 2.3 Development of the 5-stage visual rating scale

We developed a novel 5-stage visual rating scale, ranging from 0 (normal) to 4 (severe perfusion decline), to evaluate global ASL perfusion changes in the brain. This scale was formulated based on extensive observational insights from our clinical practice. We noted that as the severity of perfusion decline increases, additional areas of decreased perfusion emerge, while the decline in previously affected areas becomes more severe. Thus, higher stages on our scale incorporate the changes present in the lower stages. Intravascular ASL artifacts were identified as an important sign of perfusion decline. These artifacts are caused by the presence of marked water molecules within the arteries that have not yet perfused into the brain parenchyma, indicating a delay or reduction in perfusion [6]. This observation informed the inclusion of intravascular ASL artifacts as a key feature in our scale.

In designing our visual rating scale, we aimed to ensure that the key changes, or defining features, for each stage would be easily recognizable. While in higher stages other areas of perfusion decline may be present, the primary goal was to select hallmark changes that would enable radiologists to quickly and accurately determine the appropriate stage. By emphasizing these readily observable changes, our scale aims to streamline the assessment of global perfusion changes, making it a practical tool for use in various clinical scenarios. In our practice, we observed that it is most appropriate to evaluate the ASL stage when looking at ASL perfusion maps in a mosaic view. This allows for better visualization and comparison of perfusion patterns across different regions of the brain, facilitating the staging process.

**Fig. 1:**
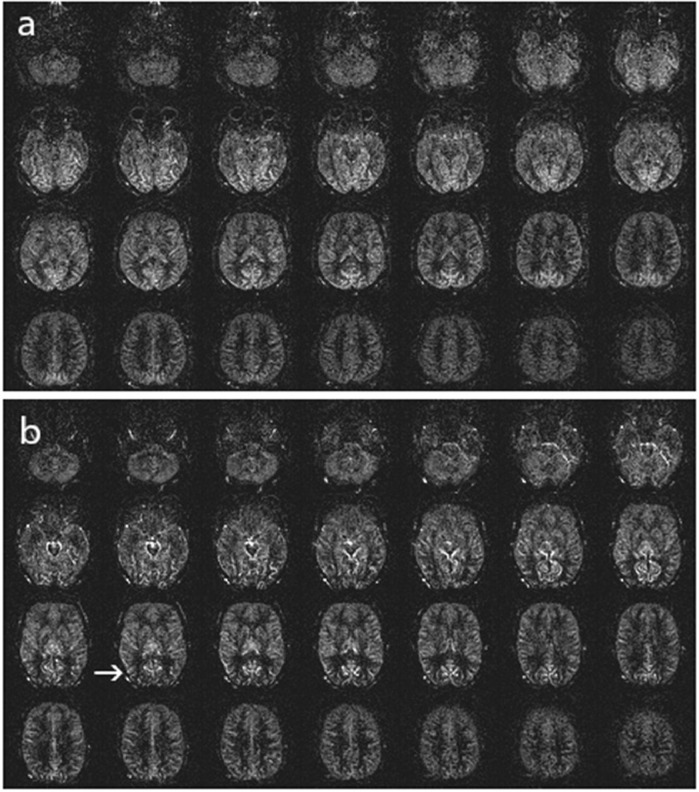
ASL Stage 0, (a): Normal ASL perfusion, (b): Slightly decreased signal in posterior watershed territories (arrow).
**Stage 1:** Either a markedly decreased signal in posterior watershed territories, the presence of ASL intravascular artifacts in middle cerebral artery (MCA) branches, or both. (Fig. 2)

The scale stages and their defining features are as follows:

**Stage 0:** Normal ASL perfusion or slightly decreased signal in posterior watershed territories.(Fig. 1)

**Fig. 2:**
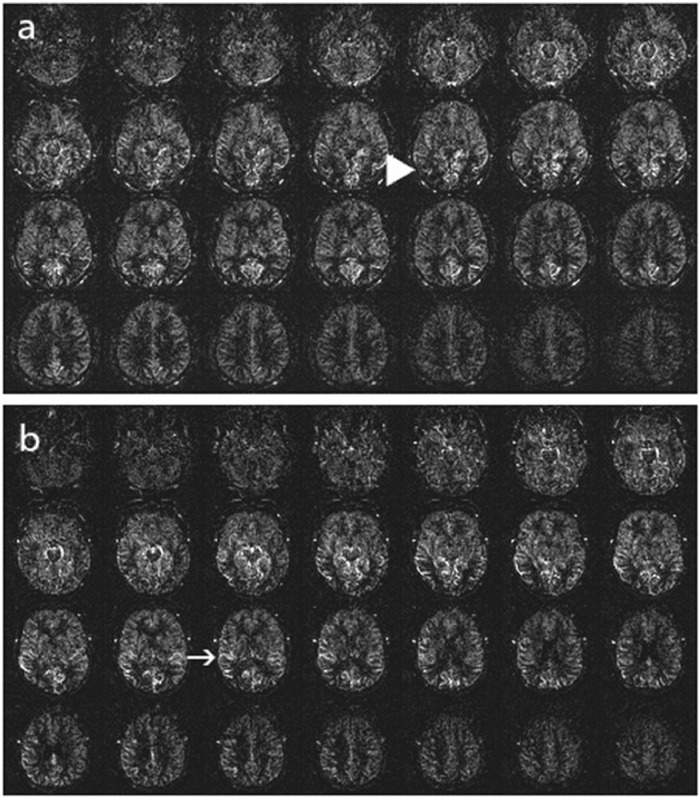
ASL Stage 1, (a): Markedly decreased signal in posterior watershed territories (big arrowhead), (b): ASL intravascular artifacts in MCA branches (arrow).
**Stage 2:** Either a moderate to markedly reduced signal in the cortex at the level of the centrum semiovale, a markedly reduced signal in anterior watershed territories, or both. (Fig. 3)

**Fig. 3:**
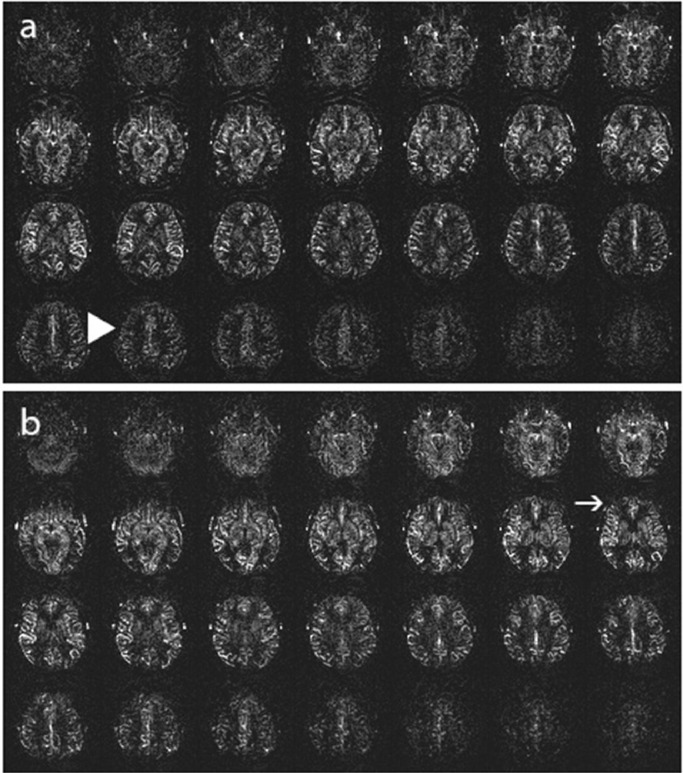
ASL Stage 2, (a): Moderately reduced signal in the cortex at the level of the centrum semiovale (big arrowhead), (b): Markedly reduced signal in anterior watershed territories (arrow).
**Stage 3:** Either a general moderate to markedly reduced signal in gray matter, numerous ASL intravascular artifacts, or both. (Fig. 4)

**Fig. 4:**
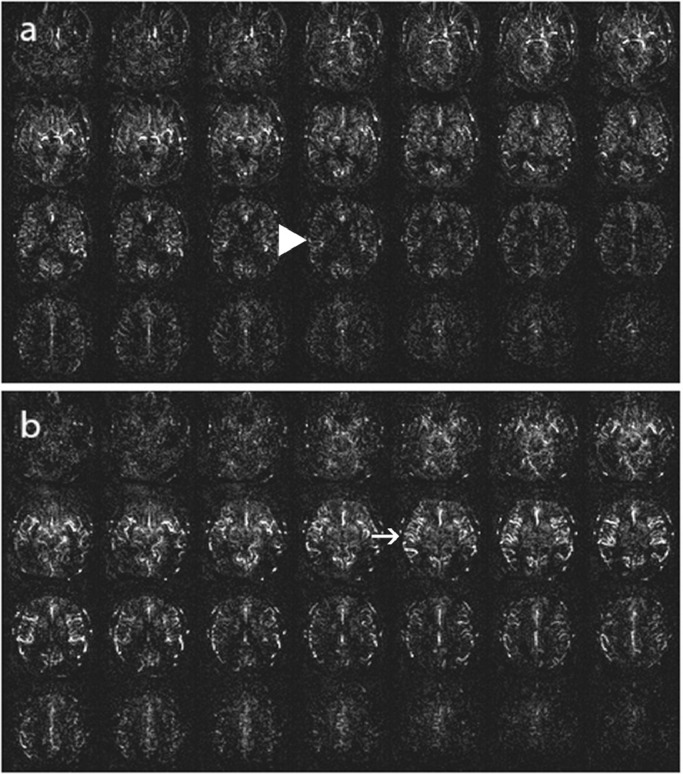
ASL Stage 3, (a): Moderately reduced signal in gray matter (big arrowhead), (b): Numerous ASL intravascular artifacts (arrow).
**Stage 4:** “ASL angio” sign - a term proposed by the authors to describe the ASL appearance at this stage. This stage is characterized by predominantly pronounced ASL intravascular artifacts, far more evident than in earlier stages, giving the image an angiogram-like appearance. Additionally, there is a notably enhanced decrease in the gray matter signal compared to previous stages, collectively indicating a severe decline in perfusion (Fig. 5).

**Fig. 5:**
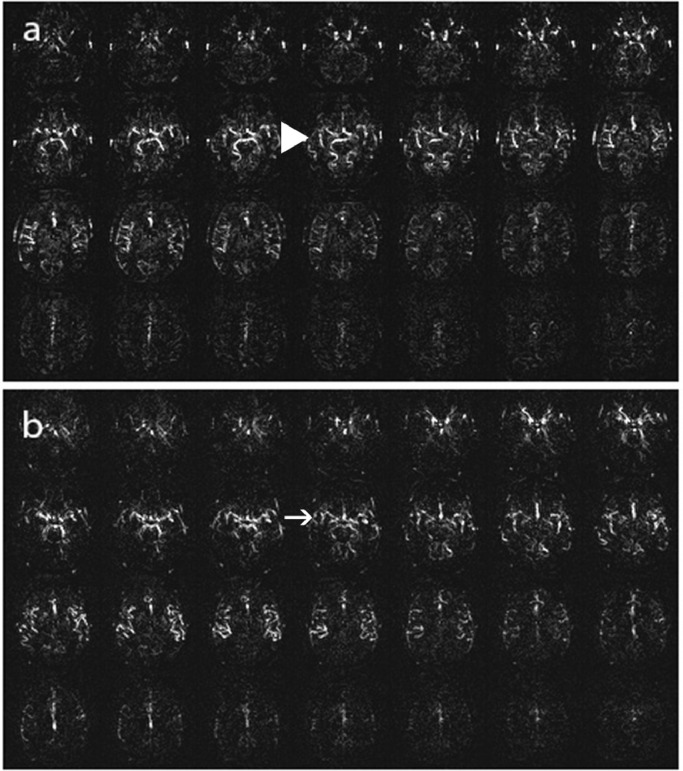
ASL Stage 4, (a): 'ASL angio' sign (ASL intravascular artifacts, giving it an angiogram-like appearance; some ASL perfusion signal may still be present in gray matter) (big arrowhead), (b): 'ASL angio' sign (arrow).

### 2.4 Interpretation of ASL data

Three radiologists with different levels of experience in ASL interpretation independently reviewed the ASL images of the 156 patients. They were blinded to each other's interpretations and assigned a stage according to the 5-stage visual rating scale. The inter-rater reliability was assessed using the intraclass correlation coefficient (ICC).

### 2.5 Statistical analysis

Descriptive statistics were used to summarize the distribution of ASL stages among the participants. In the inter-rater reliability analysis, Intraclass Correlation Coefficient (ICC) was used to assess the consistency of ASL stage ratings among the three radiologists. ICC values were calculated for both single measures and average measures, along with their respective 95% confidence intervals. All statistical analyses were performed using SPSS software, version 29 (IBM Corp., Armonk, NY, USA).

## 3 Results

### 3.1 Distribution of ASL stages

The distribution of ASL stages among the 156 patients, as determined by the three radiologists, is presented below:

Radiologist 1: Stage 0 (37.8%), Stage 1 (29.5%), Stage 2 (16.7%), Stage 3 (9.0%), Stage 4 (7.1%)
Radiologist 2: Stage 0 (34.6%), Stage 1 (32.1%), Stage 2 (17.3%), Stage 3 (9.6%), Stage 4 (6.4%)
Radiologist 3: Stage 0 (36.5%), Stage 1 (30.8%), Stage 2 (15.4%), Stage 3 (10.9%), Stage 4 (6.4%).

### 3.2 Inter-Rater reliability

The ICC analysis demonstrated high inter-rater reliability among the three radiologists. Emphasizing the consistency for individual scans, the ICC value for single measures stood at 0.947 (95% confidence interval: 0.931 - 0.959). This result highlights the strong consistency among individual ratings for each scan. In addition to this, for average measures, the ICC value was recorded at 0.982 (95% confidence interval: 0.976 - 0.986), suggesting an very high agreement when considering the mean ratings across the three raters. The F-test with true value 0 resulted in a significant p-value (<0.001) for both measures, further consolidating the reliability of the ASL staging system.

### 3.3 Clinical observations

When the 5-stage visual rating scale was applied to our study cohort, a distinct stepwise pattern in ASL perfusion was confirmed from Stage 0 to Stage 4, in line with our initial observations used for scale development. It was noted that as one progressed through the stages, the perfusion changes from the preceding stages were consistently present.

## 4 Discussion

The primary aim of this study was to develop and validate a visual rating scale for assessing global ASL perfusion changes in the brain. Our results demonstrated that the 5-stage visual rating scale showed very high inter-rater reliability among the three radiologists (ICC = 0.982), suggesting its potential utility in clinical settings. The scale also revealed a consistent stepwise decrease in ASL perfusion across stages, indicating a progressive pattern of perfusion decline.

Our visual rating scale addresses a gap in the literature, as there is no standardized scale available for evaluating global ASL perfusion changes in the brain. The simplicity of the key changes described in each stage makes it feasible for radiologists with varying levels of experience in ASL interpretation to use the scale. Furthermore, the scale's focus on global perfusion changes, rather than focal brain pathology, increases its potential applicability to various conditions that might affect the general blood supply and perfusion of the brain.

The potential applications of the 5-stage visual rating scale extend to a broad range of conditions that could alter global brain vascularization and perfusion. These conditions include:

• Cardiovascular diseases: The scale could be useful for assessing the impact of systemic cardiovascular diseases, such as heart failure, hypertension, or atrial fibrillation, on global brain perfusion [7,8,9]. Although the direct therapeutic implications of ASL-based perfusion changes remain to be elucidated, early detection might serve as a predictor for potential cerebrovascular compromise associated with these conditions.
• Cerebrovascular diseases: Patients with cerebrovascular diseases, such as stroke, transient ischemic attack, or carotid artery stenosis [4,10], could benefit from the scale's ability to evaluate global perfusion changes and monitor the progression of the disease.
• Cerebral small vessel disease (CSVD): The scale may aid in the assessment of global perfusion changes in CSVD, which is a common cause of vascular cognitive impairment and dementia [11,12,13]. Monitoring perfusion changes in CSVD could help identify patients at risk of cognitive decline and provide insights into treatment planning.
• Neurodegenerative disorders: In conditions like Alzheimer's disease and Parkinson's disease, the scale could be useful in detecting and monitoring perfusion changes related to the underlying neurodegenerative processes [4,14,15]. Early identification of perfusion changes might contribute to better understanding of disease progression.
• Visual assessment of cerebrovascular reserve: An additional consideration for future research could be the scale's applicability for evaluating the effects of modifying factors on CBF when assessing perfusion reserve [16]. Such an approach could potentially offer a simplified yet insightful way to monitor the efficacy of treatments targeting cerebral perfusion.

Our study has several limitations. First, the use of a pulsed ASL technique with a fixed inversion time and qualitative assessment could be considered a limitation. We recognize the potential constraints of this approach, particularly the reduced ability to capture the full spectrum of cerebral blood flow variability inherent in individualized inversion times. However, our decision for a fixed inversion time was intentional. By maintaining consistency, we reduce inter-scan variability, ensuring that the observed perfusion patterns are less influenced by the heterogeneity introduced by varying inversion times. This supports the primary purpose of our scale: to provide a reliable, repeatable, and simplified qualitative evaluation of global perfusion changes. These methodological choices, while potentially limiting in some contexts, simplify the interpretation process and foster the integration of the scale into everyday clinical practice. Second, our sample size of 156 patients may not be large enough to generalize our findings to a broader population. Future studies should involve larger sample sizes and diverse patient populations to confirm our results. Additionally, while we propose the visual rating scale for use in various disease states, we recognize that comprehensive empirical validation in diverse clinical settings will be essential to solidify its practical applications. Lastly, the absence of a standardized reference for ASL perfusion assessment may limit the evaluation of our scale's accuracy. However, the high inter-rater reliability observed in this study indicates that our scale is reliable and consistent among raters. Recognizing the value of comprehensive validation, we anticipate future studies to include cohorts that confirm and extend our findings.

## 5 Conclusion

**Fig. 6:**
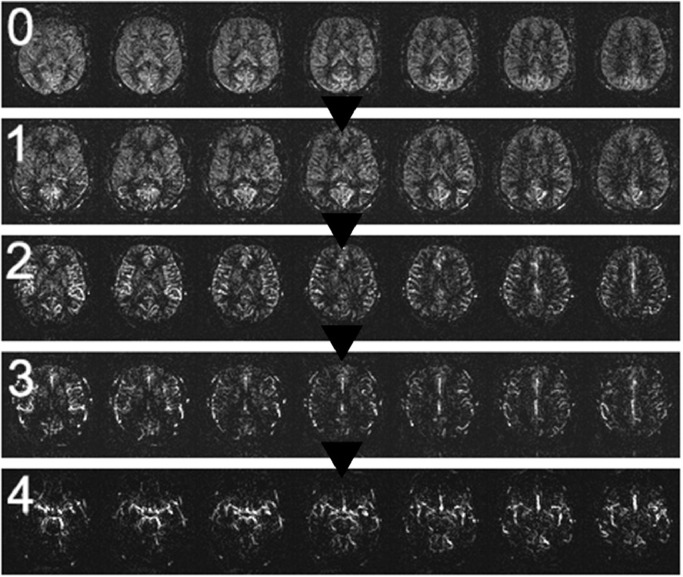
Representative images from each stage, highlighting the progression of perfusion patterns. Note: Selected images may not capture the full extent of perfusion changes typical for each stage.

We developed a 5-stage visual rating scale for global ASL perfusion changes in the brain, which exhibited very high inter-rater reliability and a consistent progressive perfusion decline pattern (Fig. 6). This practical scale has the potential to aid in assessing conditions that affect the general blood supply and perfusion in the brain. Further research is warranted to expand on its applicability.

## CRediT authorship contribution statement

**Emilian Kalchev:** Conceptualization, Data curation, Formal analysis, Investigation, Methodology, Resources, Supervision, Validation, Visualization, Writing – original draft, Writing – review & editing. **Radoslav Georgiev:** Data curation, Investigation, Resources, Validation. **Darina Ivanova:** Formal analysis, Resources, Validation.

## Declaration of Competing Interest

None.
